# The role of personality traits and moral disengagement in academic dishonesty: An analysis of the big five and the dark tetrad

**DOI:** 10.1371/journal.pone.0346573

**Published:** 2026-04-06

**Authors:** Julia Sánchez-García, Jorge Cebrián, Elena Fernández-del-Río, Pedro J. Ramos-Villagrasa

**Affiliations:** 1 Defense University Center of Zaragoza, Zaragoza, Spain; 2 Department of History and Social Sciences Applied to Design, Aragon School of Design, Zaragoza, Spain; 3 Department of Psychology and Sociology, University of Zaragoza. Faculty of Labour and Social Sciences, Zaragoza, Spain; Institute of Medical Biochemistry Leopoldo de Meis (IBqM) - Federal University of Rio de Janeiro (UFRJ), BRAZIL

## Abstract

Academic dishonesty – the inclination to cheat, falsify and plagiarise in the academic environment – is a growing concern in society, influenced by multiple psychological factors. This study aims to examine the role of personality traits (Big Five and Dark Tetrad) in academic dishonesty and exploratory analyses of potential mediating mechanisms, including of moral disengagement, in these relationships. A sample of 175 undergraduate students (*M*_*age*_ = 21.88 years, *SD*_*age*_ = 5.23) completed self-report measures, including traditional and gamified personality assessments. Results showed that extraversion, openness to experience, and responsibility traits (gamified measure) were negatively associated with academic dishonesty, while neuroticism and Dark Tetrad traits were positively related to academic dishonesty. Exploratory mediation analyses suggested that lack of moral disengagement may play a role in these personality-dishonesty relationships. These results highlight the psychological mechanisms underlying academic dishonesty behaviours, emphasising the need to examine personality not only through traditional measures, but through gamified assessments in the age of emerging technologies. Interventions targeting moral disengagement warrant further investigation.

## Introduction

According to the International Center for Academic Integrity [1], six fundamental values define academic integrity: honesty, trust, fairness, respect, conscientiousness, and courage. This article focuses on the lack of the first of these values: dishonesty. Academic dishonesty is defined as “any kind of cheating or unethical behavior in the classroom that breaches fairness and honesty principles” [2]. The prevalence of academic dishonesty among students is well documented empirically. Large-scale surveys of secondary school students indicate that academic misconduct is widespread: 64% admit to having cheated on an exam, 58% admit to having plagiarized, and 95% claim to have participated in some form of cheating [3,4]. More recently, the ICAI found, in a sample of 840 university students from multiple institutions, the continued prevalence of academic dishonesty in higher education [5]. In Europe, the prevalence of academic dishonesty is also significant. According to the European Network for Academic Integrity report [6] between 30% and 50% of European university students have admitted to plagiarism at some point in their academic life.

Evidence regarding temporal trends in academic dishonesty is mixed: longitudinal survey data suggest that plagiarism rates have not increased substantially over time [7], while recent systematic reviews indicate that the prevalence of cheating, especially in online examinations, varies by context and does not support a uniform upward trend [8]. Academic dishonesty among students indicates a lack of knowledge about academic integrity [9,10] with serious consequences for their future. For instance, Wahyuningsih et al. [11] found a positive relationship between academic dishonesty and counterproductive work behaviors. Academic dishonesty is therefore one of the major challenges facing faculty [12–14]. Strategies to foster academic integrity among students should take an educational approach [15] by solving dilemmas they are likely to face in their studies [9,10].

Given the high prevalence and negative consequences of academic dishonesty, researchers have focused on the antecedents of these behaviors. The literature on individual predictors of academic dishonesty has focused on age, gender, personality traits, and attitudes [16]. Younger students, males and those experiencing external pressures (such as high academic performance) are more likely to engage in cheating or plagiarism [17,18]. Morality is another crucial predictor. People with a high level of morality have stricter attitudes toward plagiarism because of the value they place on fairness and social rules [19]. Conversely, individuals with lower moral commitment tend to have more positive attitudes toward plagiarism [20]. In the personality domain, the Big Five model has been widely associated with academic performance and job performance. Despite its relevance, less research has been focused on the role personality traits play in academic cheating [21]. In addition, there are other personality models such as Dark Tetrad, whose traits are Machiavellianism, narcissism, psychopathy, and sadism [22,23]. These traits are characterized by the inclination to enhance one’s own status, monetary rewards, power, or superiority at the expense of others [24]. Because of the above, they are of particular interest to enhance the understanding of academic dishonesty [25].

Because of the relevance of the role of personality in the study of academic dishonesty, it is worth mentioning that the scientific evidence to date evaluates personality traits mostly through questionnaires [26]. It is known that the use of questionnaires can lead to several limitations, among which are the risk of faking, and unfavorable reactions among participants [27]. However, there are other forms of personality assessment, such as game-related assessments (GRAs), whose benefits include more favorable reactions from participants [28] and reduction of possible faking [29]. Despite its positive results, no studies are found that examine the impact of the various personality traits assessed by a gamified test on academic dishonesty. In fact, hardly any literature is found that assesses personality through a gamification process [30–32]. The present study contributes to literature by clarifying the direction of association between personality traits and academic dishonesty, as well as filling the existing gaps in terms of gamified personality assessment.

## Literature review

### Big five and academic dishonesty

Personality contemplates thoughts, feelings, and behaviors of a person with effect on academic behaviors [33]. The Big Five personality model is one of the most widely used in academic dishonesty research [34], which includes five traits: Open-mindedness to experience (O), conscientiousness (C), extraversion (E), agreeableness (A), and negative emotionality (N). In a meta-analysis, Heck et al. [35], found that the above five dimensions correlated with academic dishonesty. Specifically, the relationship of dishonesty with agreeableness and extraversion was moderate, small with open-mindedness, and ambiguous with conscientiousness and negative emotionality.

First, people with high open-mindedness to experience “actively seek out experience and tend to be particularly reflective and thoughtful about the ideas they encounter” [36]. The empirical evidence on the relationship between open-mindedness and academic dishonesty is mixed. On the one hand, some authors point to a small positive relationship between the two [37,38]; which might be due to the positive association between open-mindedness and sensation seeking [39] beyond their comfort [40]. On the other hand, most studies have indicated a small negative relationship between open-mindedness and deception [16,41–43]. One explanation is offered by Giluk and Postlethwaite [21], who indicated that open people could be less likely to cheat as they are more concerned with learning than with the grades obtained.

Second, conscientiousness refers to the predisposition to be planned, goal-directed, and to follow norms and rules [44]. In other words, it is the ability of individuals to control their own desires to focus their behavior on specific goals [45]. Those who have a high level of conscientiousness possess decisiveness, foresight, and succeed in their tasks [45]. Students who anticipate that they will not do well on a test and are highly conscientious are less likely to violate the rules against academic dishonesty [21], which supports the negative relationship between conscientiousness and academic dishonesty found by most studies [38,45,46]. However, some studies show a positive relationship between the two [40,47], therefore, further research is needed to clarify their direction.

Third, extraversion reflects the strength with which people try to interact with the social context [40]. Extraverted people tend to be sociable and assertive, while being enthusiastic and gaining energy from being around other people [48]. The thrill seeking that characterizes extroverted people provides a solid reason to link them with deception. Nevertheless, the association between extraversion and academic dishonesty is the subject of much question. While some authors point to a negative relationship between the two [33,49,50], others show a positive relationship [35], and others find no significant results [21].

Fourth, agreeableness refers to the way in which individuals deal with interpersonal relationships [40]. Kind people tend to be trustworthy, caring, and cooperative [48]. According to Giluk and Postlethwaite [21], kind students could be less inclined to cheat, copy, or plagiarize to avoid any kind of conflict. This is in line with empirical evidence so far, which supports a negative relationship between agreeableness and academic dishonesty [16,46,51].

Finally, negative emotionality refers to variations in an individual’s stability based on how they perceive the world [40]. A person with high scores on negative emotionality might interpret events as challenging or intimidating [52], and they can perceive cheating as a path to success [21], as they are more concerned about grades than learning. Relatedly, a majority of studies show a positive relationship between negative emotionality and dishonesty [21,42,43]. However, other studies show a negative relationship [53] or no relationship [50].

At this point, this research questions whether similar relationships to literature will be found by examining the Big Five through gamified assessments rather than traditional tests. In relation to this, Ramos-Villagrasa et al. [28], reported that the gamified assessment of Big Five behaved similarly to the original personality measure, therefore gamified assessments seem to have the potential to be an alternative to traditional personality questionnaires. However, there is no empirical evidence examining the relationship between the Big Five as measured by gamification and academic dishonesty, thus it is unknown whether their assessment will work as well. Furthermore, the mechanisms to explain the relationships between different personality traits and academic dishonesty require further research and understanding. The study by Guo et al. [54] showed that moral disengagement could act as a mediator in the relationship between Honesty-Humility traits and academic dishonesty. Nevertheless, how it might act with the Big Five traits is unknown.

### Dark personality and academic dishonesty

The link between dark personality traits and scholastic cheating behaviors has not yet been given sufficient attention [25]. These include narcissism, Machiavellianism, subclinical psychopathy, and everyday sadism (i.e., Dark Tetrad).

The Dark Tetrad is a personality cluster of four distinct but overlapping traits [55,56]. Narcissism is defined by entitlement, grandiosity, arrogance, self-centrism, and a sense of superiority over others [57]. Individuals high in Machiavellianism are characterized by showing a wide range of strategies to manipulate others, cynicism, cold selfishness, and impression-management tactics [58]. Those high in subclinical psychopathy are characterized by affective shallowness, impulsive thrill-seeking, and a tendency to engage in illegal and antisocial behaviors with little concern for the consequences of their acts [59]. Finally, individuals high in everyday sadism are characterized by infliction of cruelty, aggression, or humiliation on another for subjugation or pleasure [60].

Previous studies suggest that at least the three components of the Dark Triad (i.e., narcissism, Machiavellianism, and psychopathy) are positively related to academic dishonesty, with psychopathy as the strongest predictor [38,61] even after controlling other significant antecedents as cognitive ability or demographics [62]. Of the three remaining dark traits, evidence is weak for Machiavellianism [63,64] and narcissism [65], and non-existent in the case of sadism. To the best of the author’s knowledge, no study has been conducted to investigate the relationship between Dark Tetrad and the lack of academic integrity. Although psychopathy seems to be the strongest significant personality factor associated with academic dishonesty, it is important to understand the underlying mechanisms that explain this link.

Recent research indicates that the relationship between dark personality traits and academic dishonesty is often mediated or moderated by psychological factors and contextual variables. For example, among Polish university students, it was found that mastery goal orientation mediated the relationship between aspects of psychopathy (disinhibition and meanness) and academic dishonesty, and that general self-efficacy further moderated this effect [66]. Similarly, externalised responsibility mediated the association between dark triad traits and engagement in academic misconduct among students at several universities [67]. Finally, structural models based on planned behaviour theory suggest that prudence and other personality-related constructs may partially mediate the effect of personality on cheating intentions and behaviours [68]. These findings highlight that personality traits do not directly determine academic dishonesty but rather operate through complex psychological and contextual mechanisms.

One factor that may help explain academic dishonesty is moral disengagement. This concept was introduced by Bandura [69,70] to explain how humans manage to perform unethical behaviors without feeling distressed or guilty [71]. According to Detert et al. (71), moral disengagement moderates the association between individual differences (e.g., empathy, cynicism) and some unethical behaviors such as cheating. Consequently, if psychopathy implies moral deficits [72], it seems expected that moral disengagement influences the links between psychopathy and academic dishonesty [38]. However, more research is needed to explore the mediation/moderation effects [73,74] of moral disengagement in the prediction of scholastic cheating.

### Game-related assessments

Game-related assessments (GRAs) are evaluations of people’s characteristics using game elements into non-gaming contexts [32]. GRAs are becoming increasingly popular because they provide a method to measure complex constructs that reflect a test taker’s true performance level, offering potentially enhanced accountability metrics for policymakers and educators [75] with potential benefits over traditional assessments [76].

The use of GRAs instead of traditional questionnaires can enhance participant engagement, increase response rates and data quality while also reducing dishonest responses, allowing researchers to gain more authentic insights into participants’ thoughts, behaviors, or attitudes [33]. Research on GRAs has revealed that their effectiveness is shaped by design. GRAs may be derived from traditional assessments with added elements, such as challenges, game fiction, and immersion, or exist as complete games, allowing assessment methods to become progressively more playful and game-like, diverging from conventional testing formats [77]. This continuum, as proposed by Ramos-Villagrasa et al. [78] categorizes assessment methods from the more traditional to the more gamified. Thus, *gamified assessments* integrate gaming elements with traditional psychological tests to enhance user experience, increase engagement, and improve data quality through better focus and motivation. *Gamefully designed assessments* take this further by deeply embedding gamification into the assessment process, offering a more intense version. *Game-based assessments* evaluate individuals through their behavior in gaming environments, using actions like reaction times and mouse movements for analysis, diverging from traditional question-based formats. The last type of GRA is *playful games* initially designed for enjoyment, not as serious games, although some are used for assessment under certain circumstances.

There is growing research about GRAs that measure personality traits [30–32]. Harman and Brown [30] evaluated the effects of adding illustrations to game-like personality measures (GPMs) and their influence on personality assessment and participant reactions. The GPMs, both text-based and illustrated, showed significant correlations with the Big Five traits and were rated as more enjoyable and user-friendly than traditional personality inventories. Hilliard et al. [31] explored the potential of a novel, image-based personality assessment to measure the Big Five traits within recruitment contexts. Results suggest that image-based assessments can measure personality traits with accuracy comparable to traditional methods while providing shorter testing times and potentially greater engagement. Ramos-Villagrasa et al. [32] showed that measuring Big Five with a gamified version of an existing scale yields results very similar to those of the original questionnaire but improving applicant reactions. These findings highlight that gamification represents an effective and engaging alternative to traditional assessment methods. The use of GRAs to measure personality in the study of academic behavior may contribute to verifying the results of the previous literature, mainly obtained by questionnaires [41,42].

While research has focused on using gamification tests to improve students’ learning and academic integrity [9,10,79], there is no literature that assesses the relationship between the Big Five as measured by gamification and academic dishonesty. This study aims to fill this gap.

### The present study

Despite extensive research on predictors of academic dishonesty, including the Big Five traits, the Dark Tetrad traits, and moral disengagement [4], there remains a significant gap in understanding how these personality factors relate to actual dishonest behaviours as they unfold in interactive or gamified contexts. Traditional self-report measures are limited by biases such as social desirability that can obscure subtle behavioural tendencies [7,8]. GRAs offer a promising alternative, as they simulate real-life decision-making situations, capture spontaneous behaviour, and provide more ecologically valid indicators of academic dishonesty [5,80].

The study presented here provides new scientific evidence to the literature by analyzing the influence of the Big Five (open-mindedness, conscientiousness, extraversion, agreeableness and negative emotionality) on academic dishonesty using a GRA. Additionally, the influence of the Dark Tetrad traits (Machiavellianism, narcissism, psychopathy, and sadism) on dishonesty is examined. Finally, the mediating role that moral disengagement might have on the relationship between personality traits and academic dishonesty is tested.

Given previous research, the Big Five traits of open-mindedness, conscientiousness, extraversion, and agreeableness are expected to be negatively associated with academic dishonesty, except for negative emotionality, which is expected to be positively associated (*Hypothesis 1*); whereas the Dark Tetrad traits of Machiavellianism, narcissism, psychopathy, and sadism are predicted to be positively related to academic dishonesty (*Hypothesis 2*). Finally, it is hypothesized that moral disengagement will mediate the above relationships (*Hypothesis 3*). Specifically, on the one hand, high scores on the traits of open-mindedness, conscientiousness, extraversion, and agreeableness are expected to decrease moral disengagement, which in turn decreases academic dishonesty (*Hypothesis 3a*). On the other hand, high scores on negative emotionality, Machiavellianism, narcissism, psychopathy, and sadism are expected to increase moral disengagement, which in turn will increase academic dishonesty (*Hypothesis 3b*).

There is growing research about GRAs that measure personality traits [30–32]. Harman and Brown [30] evaluated the effects of adding illustrations to game-like personality measures (GPMs) and their influence on personality assessment and participant reactions. The GPMs, both text-based and illustrated, showed significant correlations with the Big Five traits and were rated as more enjoyable and user-friendly than traditional personality inventories. Hilliard et al. [31] explored the potential of a novel, image-based personality assessment to measure the Big Five traits within recruitment contexts. Results suggest that image-based assessments can measure personality traits with accuracy comparable to traditional methods while providing shorter testing times and potentially greater engagement. Ramos-Villagrasa et al. [32] showed that measuring Big Five with a gamified version of an existing scale yields results very similar to those of the original questionnaire but improving applicant reactions. These findings highlight that gamification represents an effective and engaging alternative to traditional assessment methods. The use of GRAs to measure personality in the study of academic behavior may contribute to verifying the results of the previous literature, mainly obtained by questionnaires [40,41].

## Method

### Ethics statement

Human participants were prospectively recruited for this study. Specifically, questionnaires were distributed between November 20 and 24, 2024. Prior to participation, written informed consent was obtained from all individuals involved in the study. The data used are fully anonymized and publicly available for research purposes. This research has the approval of the Research Ethics Committee of the Autonomous Community of Aragon (CEICA) (Ref. PI24/23). The dataset can be consulted in the Zenodo repository by following this link: https://doi.org/10.5281/zenodo.14870713.

### Participants

The study comprised 190 Spanish university students. Nevertheless, 13 participants were excluded because they did not respond adequately to the attention check, and two individuals were excluded from analyses in the ‘other’ gender category because their number was insufficient to form a meaningful sample. The final sample consisted of 175 participants (83% female, 17% male; *M*_age_ = 21.88 years, *SD*_age_ = 5.23) studying for bachelor’s and master’s degrees at a university different from the one to which the authors belong. Of the participants, 62.3% had work experience and 20% were currently working. A sensitivity analysis conducted with G*Power 3.1 for multiple linear regression (R² increase) indicated that, with a sample size of N = 190, the study had 80% power (α = .05) to detect an effect size of *f²* = .09. According to Cohen’s (1988) conventions, this effect size falls between small (*f²* = .02) and medium (*f²* = .15). This analysis considered all primary predictors included in the regression models (Personality traits: Big Five and Dark Tetrad; Moral disengagement; Academic dishonesty: Cheating, Plagiarism, Falsification) of academic dishonesty. For mediation analyses, which typically require larger samples to reliably detect indirect effects [81], small indirect effects may be difficult to detect with the current sample size. Accordingly, mediation results should be interpreted with caution, especially for smaller effects.

### Instruments

*Sociodemographic variables*. Participants were asked about their gender, age, and level of education.

The internal consistency (Cronbach’s α) of the following scales was calculated for the current sample:

*Academic dishonesty*. The Marsden et al. [82] scale was used, adapted to Spanish by Domínguez-Lara and Lingan-Huamán [83]. It has 14 items divided into three dimensions: cheating on exams (e.g., ‘Obtaining previous exam questions or answers’, α = .63) with 4 items, plagiarism (e.g., ‘Copying much of books or journals’, α = .80) with 5 items and, falsification (e.g., ‘Paying for previous course work’, α = .66) with 5 items. The response format followed a Likert-type scale from 1 (*never*) to 5 (*many times*).

*Big Five personality-gamified evaluation (VASSIP).* VASSIP [27] was used as a gamified version of the short version of the BFI-2-S [32,84]. It is based on storyfication, immersion, and the inclusion of game dynamics without being part of the assessment. It consists of 30 items divided into five items for each dimension identical to the original BFI-2 test: negative emotionality (e.g., ‘[I am someone who...] Worries a lot’, α = .79), extraversion (e.g.,., ‘Is full of energy’, α = .73); open-mindedness to experience (e.g., ‘Is fascinated by art, music, or literature’, α = .76); agreeableness (e.g., ‘Is compassionate, has a soft heart’, α = .64); and conscientiousness (e.g., ‘Is reliable, can always be counted on’, α = .75). The response scale follows a Likert-type format from 1 (*strongly disagree*) to 5 (*strongly agree*).

The gamified elements of VASSIP are specified in the following aspects: the storyfication consists of embedding the evaluation in a science fiction story, in which the evaluated person is hired to work in a space base whose security is compromised. During the game, the person being tested must make decisions until reaching one of the three outcomes of the story. Immersion in the test is achieved using images and music that are not part of the assessment but make it easier for the person being assessed to feel that he or she is part of a story and not part of an assessment process. Finally, VASSIP incorporates decision making and some simple games that are not part of the assessment, but help the participant perceive the experience more as a game than as an assessment.

*Dark personality*. We applied the Spanish version of the Short Dark Tetrad (SD4) [85]. This scale consists of 28 items rated on a 5-point Likert-type scale, ranging from 1 (*strongly disagree*) to 5 (*strongly agree*), with 7 items per dimension: Machiavellianism (e.g., ‘It is not wise to let people know your secrets’, α = .65), narcissism (e.g., ‘People see me as a natural leader’, α = .77), psychopathy (e.g.,., ‘People often say I am out of control’, α = .71), and sadism (e.g., ‘Watching a fist fight turns me on,’ α = .73).

*Moral disengagement*. The 8-item scale developed by Moore et al. [86] was used, using the translation by Ramos-Villagrasa et al. [87]. It is rated on a 7-point Likert scale ranging from 1 (*strongly disagree*) to 7 (*strongly agree*). The observed reliability is 0.70. A sample item is ‘It’s okay to spread rumors to defend those you care about.

Internal consistency (Cronbach’s α) was calculated for the current sample. Several scales showed modest reliability (α = 0.63–0.66). While these values are slightly lower than ideal, they are within acceptable ranges for research with brief personality and attitudinal measures. Lower reliability may attenuate associations in regression and mediation analyses, potentially underestimating effect sizes. Consequently, results are interpreted cautiously, emphasizing converging patterns across multiple measures.

### Procedure

The students were recruited from a university different from that of the research team, with the collaboration of professors in the same field (i.e., Work and Organizational Psychology). These professors invited the students to participate in a supplementary session within their current courses, where they would have the opportunity to play a serious game and receive a feedback report on their personality. The sessions offered no monetary incentives and had no impact on students’ grades. Members of the research team were present only to verbally explain the research purposes and participant rights—in accordance with the ethical standards of the American Psychological Association (APA)—and to address any technical issues. Following the briefing, participants were provided with a link to a 20-minute self-report questionnaire where they gave informed consent and completed measures on sociodemographic data, dark personality, and academic dishonesty. Subsequently, they accessed VASSIP for approximately 40 minutes to complete the personality measure based on the Big Five model.

### Analysis

The analyses included reliability analysis, descriptive statistics, correlations, regressions, and exploratory mediation analyses. All analyses were performed with the IBM SPSS v29.0 statistical program. Subsequently, mediation analyses were conducted using Model 4 through the PROCESS macro of SPSS v29.0 using a bootstrapping procedure with 5,000 replicates [88]. Linear regression was applied to ordinal academic dishonesty scales. This approach is widely accepted for Likert-type scales with five or more points, where deviations from interval-level measurement minimally affect estimates [89]. No covariates were included in the mediation models, as the analyses focused on examining the direct and indirect associations among the primary study variables. In addition, to maintain sufficient statistical power and avoid overparameterization across 27 mediation tests given the sample size (N ≈ 160–190), potential covariates such as age and sex were not included. Potential confounding effects should be considered when interpreting the results.

Scale scores were computed using mean scores rather than total scores. Mean scores retain the original response metric of the items, facilitating interpretation of unstandardized regression coefficients and comparability across scales with different numbers of items. As noted in the psychometric literature [90], means and sums are linearly equivalent when all data are complete, but means preserve the measurement units of the original items, which enhances interpretability in multivariate analyses.

A post hoc power analysis was conducted for each mediation model using WebPower (simple mediation using Sobel’s test). For the observed sample size and effect sizes of paths a and b, the power to detect the indirect effect was between 0.17 and 0.43, indicating limited power to detect small indirect effects. No correction for multiple comparisons was applied as all mediation analyses were exploratory (post-hoc power = 0.17–0.43). Therefore, all mediation results should be interpreted with caution due to inflated Type I error risk and considered exploratory.

## Results

### Descriptive statistics and assumption checks

Prior to hypothesis testing, the assumptions for regression and mediation analyses were examined. Visual inspection of standardized residuals and predicted values (histograms, Q–Q plots, and scatterplots of standardized residuals against standardized predicted values) indicated approximately normal distributions across all models, with no substantial deviations from normality. Linearity and homoscedasticity were supported by scatterplots of residuals versus predicted values. Minor positive asymmetry was observed in some cases. Durbin–Watson statistics ranged from 1.73 to 2.09, indicating no evidence of autocorrelation of residuals. Multicollinearity was not a concern in any model, as all variance inflation factor (VIF) values were below 5 and tolerance values exceeded.20.

Descriptive statistics (means, standard deviations, skewness, and kurtosis) were performed to determine the characteristics of the sample. Table 1 presents the results for all variables. Continuous variables were examined for normality using skewness and kurtosis. Continuous variables generally showed distributions close to normality, with the exceptions of age and falsification. Age was positively skewed and leptokurtic, which may reflect the restricted range of the sample (i.e., university students). The falsification variable also showed positive skewness and leptokurtosis, indicating that most participants selected responses near the lower end of the scale. Due to the above, correlations among study variables were estimated using Spearman rank-order correlations to provide a consistent and robust approach.

**Table 1:** Descriptive statistics for variables.

Variables	*M*	*SD*	Skewness	Kurtosis
Gender	0.17	0.38	1.77	1.13
Age	21.88	5.23	4.17	20.91
Cheating in exams	2.16	0.75	0.29	−0.66
Plagiarism	2.32	0.85	0.69	−0.25
Falsification	1.48	0.54	1.72	3.89
Open-Mindedness	3.90	0.64	−0.25	−0.80
Conscientiousness	3.70	0.69	−0.21	−0.83
Extraversion	3.61	0.64	−0.66	0.79
Agreeableness	3.99	0.55	−0.46	−0.16
Negative Emotionality	2.97	0.64	0.17	0.18
Machiavellianism	2.92	0.62	−0.23	−0.30
Narcissism	2.81	0.74	−0.20	−0.78
Psychopathy	1.67	0.56	0.98	0.66
Sadism	1.91	0.67	0.59	−0.43
Moral disengagement	2.13	0.64	0.27	−0.52

Regarding personality traits, the highest scores were observed in agreeableness and open-mindedness, while the lowest scores appeared in negative emotionality. For dark personality traits, participants scored higher on Machiavellianism and narcissism compared to psychopathy and sadism. Among types of academic dishonesty, plagiarism was more prevalent than other forms.

The variable gender is categorical (coded as 0/1), and therefore measures of central tendency, standard deviation, skewness, and kurtosis are not meaningful; instead, frequencies and percentages should be reported.

### Correlation analysis

Spearman correlations were used to examine the associations between the study variables. Spearman rank correlations were used due to non-normality in age and falsification scales (Shapiro-Wilk p < .001). Supplementary Pearson correlations produced substantively equivalent results (mean absolute difference |r-ρ| ≈ 0.02; see Table in S1 Table). As for the effects of demographic variables on indicators of academic dishonesty, age correlated negatively only with plagiarism. However, this association should be interpreted cautiously, as the sample’s age distribution is restricted and positively skewed, consisting solely of university students. Therefore, while the correlation is statistically significant, it may not generalize to a broader population. No significant association was found for gender as can be seen in Table 2.

**Table 2:** Results by correlational analysis.

Variables	1	2	3	4	5	6	7	8	9	10	11	12	13	14	15
1. Gender															
2. Age	.15*														
3. Cheating in exams	.11	−.12													
4. Plagiarism	.07	−.28***	.45***												
5. Falsification	.14	−.11	.45***	.44***											
6. Open-Mindedness	−.03	.01	−.02	−.15	−.03										
7. Conscientiousness	−.14	.15	−.08	−.23**	−.23**	−.04									
8. Extraversion	−.02	.22**	−.13	−.21**	−.14	.34***	.25***								
9. Agreeableness	−.10	.14	−.06	−.19*	−.09	.11	.15*	.11							
10. Negative Emotionality	−.10	−.18**	−.05	.04	−.03	.00	−.18**	−.08	−.25***						
11. Machiavellianism	.21**	−.19**	.22**	.24**	.16*	.01	−.05	−.05	−.13	−.11					
12. Narcissism	.22**	.02	.14	.05	.14	.36***	.00	.37***	.03	−.22***	.28***				
13. Psychopathy	.27***	−.09	.22**	.30***	.24**	.11	.30***	.07	−.29***	−.07	.26***	.38***			
14. Sadism	.35***	−.09	.28***	.33***	.32***	.01	−.25***	−.11	−.30***	−.07	.46***	.33***	.42***		
15. Moral disengagement	.14	−.16*	.28***	.30***	.16*	−.27***	−.11	−.25***	−.26***	.18**	.36***	.07	.29***	.43***	

*Note*: *p < .05, **p < .01, ***p < .001.

Regarding the effects of Big Five personality traits on academic dishonesty, it was found that plagiarism is related to three traits: Extraversion, agreeableness, and conscientiousness, indicating that higher scores in these traits are associated with lower levels of plagiarism. The dimension falsification was negatively associated with conscientiousness, showing that participants with higher conscientiousness reported lower levels of falsification. Cheating on exams showed no significant correlations with any Big Five trait.

Dark Tetrad was also linked with academic dishonesty: plagiarism was positively associated with psychopathy and sadism, suggesting that higher levels of these traits are associated with higher levels of plagiarism. Falsification was positively associated with Machiavellianism, psychopathy, and sadism; while cheating on exams showed a similar positive pattern.

Concerning the effect of moral disengagement on academic dishonesty, moral disengagement was positively associated with cheating, plagiarism, and falsification, indicating that higher moral disengagement scores are associated with higher engagement in dishonest behaviors. Some variables, such as age, have a restricted and skewed distribution, so these correlations reflect patterns within this sample rather than generalizable population trends.

### Regression analysis

Linear regressions were used to examine the influence of various personality traits and moral disengagement on academic dishonesty through four models. Model 1 introduces sociodemographic variables (gender, age, and education level). Model 2 includes the Big Five personality traits, while Model 3 adds the Dark Tetrad traits. Finally, Model 4 introduces moral disengagement. The coefficients of the variables considered in each model remained stable as a sign of robustness of the results Table 3 shows the estimates of Model 4 of academic dishonesty (see supplementary material for Models 1, 2 and 3 in S2 Table, S3 Table and S4 Table).

**Table 3:** Unstandardised regression coefficients of academic dishonestity on tests predicted by socio-demographics, personality and moral disengagement: Model 4.

		Cheating			Plagiarism			Falsification	
*Predictors*	*b(SE)*	*95% CI*	*p*	*b(SE)*	*95% CI*	*p*	*b(SE)*	*95% CI*	*p*
Gender	−.02(.17)	−.37−.30	.84	−.11(.17)	−.58−.10	.16	−.10(.12)	−.37−.10	.21
Age	−.02(.01)	−.03−.02	.79	−.03(.01)	−.03−.02	.69	−.00(.01)	−.02−.02	.96
Education level	−.01(.04)	−.08−.07	.90	−.01(.04)	−.09−.07	.84	−.04(.03)	−.07−.04	.60
Open-Mindedness	.01(.11)	−.20−.22	.94	−.17(.11)	−.44−.01	**.04**	−.11(.07)	−.23−.05	.21
Conscientiousness	.07(.10)	−.12−.26	.45	−.00(.10)	−.20−.19	.99	−.00(.07)	−.14−.13	.95
Extraversion	−.16(.11)	−.40−.03	.08	−.19(.11)	−.48−.03	**.02**	−.19(.07)	−.31−.01	**.03**
Agreeableness	.08(.12)	−.12−.35	.35	.02(.12)	−.21−.27	.82	.08(.08)	−.08−.24	.32
Negative Emotionality	−.02(.10)	−.23−.17	.79	.18(.09)	.03−.38	**.02**	.11(.06)	−.04−.20	.19
Machiavelianism	.07(.10)	−.22−.22	.97	.10(.11)	−.09−.37	.23	.04(.08)	−.12−.18	.67
Narcissism	.00(.11)	−.13−.27	.50	.10(.11)	−.10−.32	.30	.15(.07)	−.03−.25	.12
Psychopathy	.17(.13)	−.03−.48	.08	.27(.13)	−.14−.66	.**00**	.19(.09)	.01−.36	.**04**
Sadism	.15(.12)	−.07−.40	.16	.15(.12)	−.05−.42	.11	.27(.08)	.06−.38	.**01**
Moral disengagement	.11(.12)	−.10−.36	.26	−.01(.12)	−.25−.22	.90	−.10(.08)	−.24−.08	.31
*R*^*2*^*/ R*^*2*^ *adjusted*	.13/.05			.28/.22			.20/.13		

*Note*. *b* = beta; *SE* = Standard Error; *95% CI* = Confidence Interval, *p* = p value.

In Model 4, the results showed two significant regressions on two types of academic dishonesty: plagiarism [*F*(13, 150) = 4.50, *p* < .001], and falsification [*F*(13, 150) = 2.92, *p* < .001]. The *R*^*2*^ was.28 for plagiarism and.20 for falsification, indicating that the various personality traits and moral disengagement explained approximately 28% of the variance in committing plagiarism, and 20% in falsifying a document. Specifically, extraversion and open- mindedness reduce the probability of committing plagiarism; while negative emotionality and psychopathy increase it. Extraversion also decreased the probability of falsification, whereas psychopathy and sadism increased it. However, no significant data was obtained for moral disengagement.

As for cheating on exams, Model 3 showed a marginally significant negative association for extraversion (*b* = –0.18, *SE* = 0.11, *t* = –1.97, *p* < .05), indicating that higher extraversion tended to decrease the probability of cheating. The association with psychopathy was also marginally significant (*b* = 0.19, *SE* = 0.13, *t* = 1.97, *p* = .05), bu*t* the 95% confidence interval included zero (–0.00 to 0.50), suggesting that this effect should be interpreted cautiously. These effects were no longer significant in Model 4 [*F* (13, 150) = 1.68, *p* = .07] after introducing moral disengagement. Given the marginal effect sizes in Model 3, the disappearance of significance likely reflects the limited stability of these associations combined with the additional variance explained by moral disengagement, rather than issues of multicollinearity (VIFs < 5; tolerance > .20).

### Mediation analyses

Finally, because the introduction of moral disengagement increased the variance explained for each indicator of academic dishonesty, exploratory mediation analyses were conducted to assess whether moral disengagement mediated the relationship between the various personality traits and academic dishonesty (9 traits x 3 dimensions of academic dishonesty = 27 mediation analyses). The analyses were conducted using the bootstrapping procedure with 5,000 samples to estimate confidence intervals for indirect effects. Post-hoc power analyses indicated limited power to detect small indirect effects. For reasons of space, only the significant spillover effects are mentioned, although the repository mentioned in the Method section contains all the analyses conducted and the database for replication.

Regarding the Big Five personality traits, the analysis showed two significant indirect effects for extraversion on cheating (*β* = −.06, 95% CI [−.13, −.02]) and plagiarism (*β* = −.09, 95% CI [−.16, −.02]) across moral disengagement; three significant indirect effects for agreeableness on cheating (*β* = −.09, 95% CI [−.17, −.02]), plagiarism (*β* = −.11, 95% CI [-. 22, −.03]), and falsifying (*β* = −.04, 95% CI [−.10, −.00]); two significant indirect effects for negative emotionality on cheating (*β* = .04, 95% CI [.01,.10]), and plagiarizing (*β* = .06, 95% CI [.01,.12]); and, three significant indirect effects for open-mindedness to cheating (*β* = −.09, 95% CI [−.18, −.03]), plagiarizing (*β* = −.11, 95% CI [−.22, −.04]), and, falsifying information (*β* = −.04, 95% CI [−.10, −.00]). Nevertheless, no significant indirect effect was obtained for the trait of conscientiousness. This suggests that extraversion, agreeableness and open-mindedness decrease moral disengagement, which in turn reduces academic dishonesty. Conversely, Negative emotionality increases moral disengagement, which increases academic dishonesty.

Finally, dark personality traits showed several significant indirect effects for cheating on tests and plagiarism, except for falsifying. Specifically, the Machiavellianism trait showed two significant indirect effects on cheating (*β* = .09, 95% CI [.02,.17]) and plagiarizing (*β* = .12, 95% CI [.04,.22]); trait psychopathy found two other significant indirect effects on cheating (*β* = .07, 95% CI [.01,.16]) and plagiarism (*β* = .10, 95% CI [.02,.22]); and a significant indirect effect of trait sadism on plagiarism (*β* = .11, 95% CI [.02,.22]). However, no significant indirect effect was obtained for trait narcissism. These preliminary patterns suggest that Machiavellianism, psychopathy, and sadism traits are associated with higher levels of academic dishonesty via increased moral disengagement, consistent with theoretical expectations. However, given the low statistical power and multiple testing, these findings are exploratory and require replication in larger samples (see Discussion).

The inclusion of moral disengagement as a mediator is grounded in social-cognitive theory, which conceptualizes moral disengagement as an active self-regulatory process that enables individuals to decouple internal moral standards from behavior, thereby facilitating unethical actions [69,70]. Empirical evidence shows that moral disengagement mediates the relationship between personal characteristics and unethical conduct across domains [71] and specifically in academic contexts where it mediates the influence of personality traits on academic dishonesty [91]. Additionally, research on cyberbullying and aggression models further supports its mediating role between individual differences and behavioral outcomes.

## Discussion

The study reported in this paper contributes to the nomological network of academic dishonesty in four different ways: (1) Verify previous research on the relationship between the Big Five and academic dishonesty using a different assessment method (i.e., gamification); (2) incorporate the Dark Tetrad into the study of academic dishonesty, highlighting the relevance of sadism in explaining these counterproductive behaviors; (3) Provides exploratory evidence that different personality traits predict distinct academic dishonesty dimensions (cheating, plagiarism, falsifying), with moral disengagement emerging as a potential mediator in preliminary analyses (power = 0.43–0.17 across 27 tests). Notably, this is the first study to demonstrate convergent validity between gamified and traditional personality assessments in predicting academic dishonesty, highlighting the promise of gamification for personality measurement in educational contexts.

These findings could be of interest for the achievement of the United Nations Sustainable Development Goals (SDG), specifically Goal 4: *Quality Education*. Understanding the relationship between personality and academic dishonesty can inform discussions on promoting a culture of honesty in academic contexts, and contribute to understanding factors related to dishonest behaviors in learning and potentially in later professional settings.

### The big five and academic dishonesty

The first contribution of this study was to show the relationship between the Big Five and academic dishonesty using a gamified assessment. This approach illustrates the use of gamified assessments in measuring personality traits. The results show that extraversion, open-mindedness to experience and conscientiousness are negatively related to some dishonest behaviors; while negative emotionality is positively related to plagiarism, in relation to research Hypothesis 1. These results are in line with findings from the literature [33,50]. Extraversion was the personality trait that showed the strongest negative association with academic dishonesty, clarifying the ambiguous evidence so far [16,21,35]. Extraverts could avoid procrastination due to their enjoyment of multitasking [92], as well as their high resilience associated with high energy and productivity [93]. Open-mindedness to experience was the second Big Five personality trait to be associated with academic dishonesty. Individuals who score high on open-mindedness tend to be interested in complex problems, which is associated with higher academic achievement [94]; therefore, they might be less interested in plagiarizing information. It is possible that individuals high in conscientiousness are less academically dishonest due to their high level of self-discipline, organization, goal orientation, and respect for norms and rules [95]. In contrast, individuals high in negative emotionality are more likely to engage in plagiarism, which may be due to their impulsivity, volatility, test anxiety, and even their focus on performance to the detriment of their own learning [16].

### Dark tetrad and academic dishonesty

Consistent with Hypothesis 2, Machiavellianism, psychopathy, and sadism were positively related to academic dishonesty. These findings are in line with prior research [40,61,63,64] where correlations were moderate. Machiavellianism may be associated with cheating, plagiarism, and falsification as instrumental strategies aimed at achieving self-interested goals, such as maintaining a favorable academic image [58]. As Williams et al. [40] pointed out, these individuals may be more selective when engaging in certain forms of dishonest behavior (e.g., plagiarism instead impulsive cheating) than psychopathy and sadism, which have been linked in prior research to higher levels of impulsivity. However, results of regression analysis indicated that only psychopathy was a significant predictor of plagiarism and falsification as warned by previous studies [61,62]. Students with higher levels of psychopathy may be less sensitive to contextual influences, as suggested by prior research [63]. Importantly, the interpretation of these findings requires caution. Although the Discussion refers to behavioral tendencies such as impulsivity, thrill-seeking, or ego-related motivations when interpreting associations between Dark Tetrad traits and academic dishonesty, these mechanisms were not directly assessed in the present study. Therefore, such interpretations should be understood as theoretically informed inferences rather than empirically tested processes within this dataset.

In this regard, it is particularly relevant that regression analyses indicated that only psychopathy remained a significant predictor of plagiarism and falsification when controlling for the other dark traits. This finding is consistent with some prior studies identifying psychopathy as the most robust dark personality correlate of dishonest or counterproductive behaviors, but it also contrasts with other research reporting mixed or trait-specific effects. One possible explanation is that psychopathy captures core dispositional features—such as low impulse control, reduced sensitivity to sanctions, and diminished moral concern—that are more proximally linked to enacted dishonest behavior, whereas traits such as Machiavellianism, narcissism, or sadism may exert their influence indirectly or contingently on contextual or motivational factors.

Moreover, the use of the SD4 instrument, while advantageous for brevity and feasibility, entails certain psychometric limitations. In particular, the narcissism subscale primarily captures grandiose aspects of narcissism and does not assess other theoretically relevant dimensions (e.g., vulnerable narcissism or exhibitionism), which may partly account for the absence of significant effects in the regression models. Consequently, null findings for narcissism—and weaker effects for other dark traits—should not be interpreted as definitive evidence of non-association but rather as reflecting measurement constraints and the complexity of these constructs.

Overall, these results underscore the need for future research to incorporate more comprehensive assessments of dark personality traits and to directly measure mediating mechanisms such as impulsivity, moral cognition, or motivational orientations. Such approaches would allow for a more precise disentanglement of the pathways through which different dark traits contribute to academic dishonesty.

Beyond these findings, the association observed between sadism and academic dishonesty warrants further theoretical consideration. To clarify the nature of this association, it is important to distinguish everyday sadism from overt interpersonal aggression. Although everyday sadism has traditionally been examined in relation to aggression and direct harm to others [96], recent research conceptualizes it more broadly as a dispositional tendency toward enjoying or tolerating norm violations, domination, and transgressive acts [55] From this perspective, academic dishonesty may represent a low-risk context in which individuals high in sadism can express rule-breaking tendencies without immediate social sanctions.

Importantly, plagiarism and cheating do not require direct interpersonal confrontation, but they do involve the violation of institutional norms and moral standards. Individuals high in sadism may therefore be less constrained by such standards, potentially due to diminished moral concern and a greater willingness to disengage moral self-regulation. This interpretation is supported by the observed indirect effects through moral disengagement, suggesting that the association between sadism and academic dishonesty is primarily moral-cognitive rather than aggressive in nature. Findings from organizational research provide convergent support for this interpretation. For example, Fernández-del-Río et al. [97] found that sadism was the most important predictor of counterproductive work behaviors (CWBs) that resembles counterproductive academic behaviors but in the industrial and organizational context. According to Salgado et al. [98], both types of behaviors are intentional, violate significant institutional norms, damage the institution, its members, or both, and that lead to several undesirable consequences for the organization and others. Therefore, even when more studies are needed, our study provides initial evidence supporting the relevance of sadism in the prediction of certain forms of academic dishonesty (i.e., plagiarism).

Taken together, this interpretation highlights the importance of considering moral-cognitive mechanisms when examining the role of dark personality traits in academic dishonesty, while also underscoring that not all dark traits operate in the same way.

Contrary to our hypothesis and most of previous literature, narcissism was not associated with academic dishonesty. Some plausible explanations can be offered. First, it could be due to the overlap with other dark personality traits [38]. Second, prior research suggests that narcissistic individuals’ performance may be more sensitive to ego-relevant contextual cues [99], so if it could be threatened, for example for the publicizing of exam results, they would be enhanced to perform academically dishonest behaviors. If not, they would not risk being punished if plagiarism is detected or to be ashamed if they are caught cheating during an exam. Third, narcissism items of SD4 captured only grandiose narcissism, whereas other dimensions (i.e., exhibitionism factor) are not considered. As Brunell et al. [100] found, it seems that the role of specific dimensions of narcissism or the mechanism behind these effects might be considered when we explore the link between narcissism and academic dishonesty.

### Mediation of moral disengagement

Regarding Hypothesis 3a, exploratory analyses suggested that extraversion, agreeableness and open-mindedness were associated with lower moral disengagement, which in turn was associated with lower levels of academically dishonest behaviors. These tentative patterns may clarify the mixed or inconsistent findings so far on the relationship between the Big Five and moral disengagement [101–104], though causal inferences are precluded by the cross-sectional design. The negative association between extraversion and academic dishonesty through low moral disengagement may reflect extraverted individuals’ enthusiasm for positive social interactions [105]. For open-mindedness, this association could be explained by the intellectual component of the trait, which may facilitate abstract reflection and idea development [106], potentially leading to lower moral disengagement and, consequently, fewer dishonest behaviors.

The negative relationship between agreeableness and academic dishonesty through lower moral disengagement aligns with Egan et al. [103], as agreeableness addresses reflects politeness and respect for social norms, as well as recognition of others desires and goals. Negative emotionality showed the clearest positive indirect pattern with academic dishonesty via moral disengagement, potentially reflecting poor impulse control and irritability, promoting moral disengagement [106].

No indirect pattern emerged for conscientiousness, possibly due to the specific indicators of academically dishonest behaviors employed in this study, which differ from those used in prior research focusing on workplace dishonesty [107]. Additionally, conscientious individuals are generally characterized by self-discipline, rule-following, and a strong sense of responsibility [108], which may reduce the variability in moral disengagement and limit its mediating role in this sample. Thus, conscientiousness may influence academic dishonesty through alternative mechanisms. Some findings show that more specific constructs such as impulsivity and neutralization strategies often explain more variance in dishonest conduct than broad personality domains, suggesting that conscientiousness may influence unethical behavior through mechanisms other than moral disengagement per se (e.g., self-control, planning, task commitment) [16].

On the other hand, negative emotionality, Machiavellianism, narcissism, psychopathy, and sadism were associated with higher levels of moral disengagement, which in turn was linked to increased academic dishonesty. These preliminary findings suggest the moral disengagement was particularly supported for Machiavellianism, psychopathy, and sadism, although the findings suggest that different components of the Dark Tetrad may operate differently across types of dishonest behaviors. As prior literature pointed out [109], students engaging in academically dishonest behaviors tend to employ cognitive strategies to justify or rationalize actions that violate university norms. For example, Machiavellians individuals could resolve moral conflicts arising from cheating or plagiarism by appealing to outcome-based justifications (i.e., “the end justifies the means”). Prior research has linked psychopathy to impulsivity and reduced moral inhibition, which may facilitate academically dishonest behaviors [38].

### Theorical and practical implications

Personality assessment in the research of academic behavior has primarily relied on traditional questionnaires. The integration of game elements into personality assessment has been demonstrated to replicate real-world decision-making processes, thereby providing valuable insights into how individuals navigate ethical dilemmas in dynamic, immersive environments [77]. This study demonstrates that gamified assessments serve as a novel and effective alternative to traditional methods for measuring personality traits in the context of academic behavior.

The adoption of GRAs could enhance the validity and reliability of research findings across diverse fields, potentially transforming data-collection methodologies in both academic and professional contexts. The integration of gamified assessments within university settings presents a promising approach to the identification of tendencies towards academic dishonesty, thereby contributing to the development of a more ethical academic environment [9,10].

By understanding personality traits, universities can provide more targeted and effective support to help students achieve academic success. Using personality tests to tailor academic support for students involves focusing on three key traits: open-mindedness, conscientiousness and agreeableness [33]. Open-mindedness reflects how receptive someone is to new ideas and experiences, conscientiousness measures how organized, responsible and hardworking a person is, and agreeableness indicates how cooperative and friendly a person is. Identifying students who score low on these traits can help schools provide targeted support. For example, students with low open-mindedness might benefit from extra tutoring to help them understand new concepts. Those with low conscientiousness could improve their organizational skills through time management workshops. Students with low agreeableness might need resources to help reduce test anxiety, as they may have more difficulty coping with stress in an academic setting.

Universities could leverage these tools not only to enhance integrity policies but also to design targeted support programs for specific student profiles. For instance, students who frequently experience worry or anxiety, demonstrating elevated levels of Negative emotionality, and students who exhibit dark personality traits, such as manipulation, characteristic Machiavellianism, a lack of empathy or callousness, as seen in psychopathy, or a propensity to inflict harm upon others, which is associated with sadism, are particularly vulnerable [64]. These programs would focus on improving personal growth and behavior by teaching emotional management strategies, fostering a stronger sense of morality, and providing training on handling disagreements and problems with others more effectively. The goal would be to help these students develop more positive behaviors and effective coping strategies.

### Limitations

The present study has several limitations that should be considered. A primary constraint pertains to the sample size and composition, with the study encompassing 175 university students, a relatively modest sample size that curtails the generalizability of the findings. Moreover, the preponderance of a youthful, Spanish demographic within the sample diminishes the applicability of the conclusions to alternative age groups, educational contexts, or cultural milieus. Another limitation is the measurement of academic dishonesty, which relied on self-reporting, although the use of gamified assessments aimed to mitigate the risk of response bias. However, the sincerity of participants’ responses could still have been influenced by social desirability or fear of judgment. Several scales showed moderate reliability (α = 0.63–0.66). Although these values are slightly below ideal, they are within acceptable ranges for research with brief personality and attitude measures [110]. Lower reliability may attenuate associations in regression and mediation analyses, which could lead to underestimating the effect size. Therefore, the results are interpreted with caution, emphasizing convergent patterns across multiple measures.

Regarding mediation analyses, although bootstrapping was used to estimate confidence intervals for indirect effects, these effects were relatively small and the analyses involved multiple mediation tests without adjustment for multiple comparisons. In combination with the modest sample size, this may limit the precision and generalizability of the findings. Therefore, the mediation results should be interpreted as preliminary and indicative of potential pathways rather than definitive evidence of causal mechanisms. Specifically, although mediation was a central component of the hypotheses, post hoc power analysis indicated that the study had limited power to detect small indirect effects. Therefore, the mediation results should be considered exploratory and require replication in larger samples before firm conclusions about indirect pathways can be drawn.

A further limitation is the cross-sectional nature of study. Longitudinal studies would provide a deeper understanding of how these variables interact and evolve. Finally, the study’s reliance on specific personality models, such as the Big Five and the Dark Tetrad, restricts the study’s analytical scope. While these models are highly relevant, they exclude other potential predictors of academic dishonesty, such as academic motivation or socioeconomic factors. SACCIA (Sufficient, Accurate, Clear, Contextualised and Interpersonally Adaptive) communication could also be examined, as research shows that communication can reduce academic misconduct by reducing positive attitudes toward cheating; however, it is unknown whether such communication mediates the relationship between personality traits and academic dishonesty [111]. Incorporating these variables in future research endeavors would facilitate a more comprehensive understanding of the factors that contribute to dishonest behaviors in academic settings. Finally, although gamified assessments were used in this study, social desirability bias was not directly measured or controlled for. Therefore, it cannot be empirically concluded that gamification reduces socially desirable responses. Future research should explicitly assess social desirability to determine whether gamified formats can effectively mitigate this potential bias.

Addressing these limitations would allow for the development of more robust and widely applicable insights into the relationship between personality traits and academic dishonesty.

## Supporting information

S1 Table Complete Pearson Correlation Matrix. (DOCX)

S2 Table Unstandardised regression coefficients of cheating on tests predicted by socio-demographics, personality and moral disengagement. (DOCX)

S3 Table Unstandardised regression coefficients of plagiarism predicted by socio-demographics, personality and moral disengagement. (DOCX)

S4 Table Unstandardised regression coefficients of falsification predicted by socio-demographics, personality and moral disengagement. (DOCX)
