# Supplementary material for: The role of personality traits and moral disengagement in academic dishonesty: An analysis of the big five and the dark tetrad
Source: PLoS One. 2026 Apr 6;21(4):e0346573. doi: 10.1371/journal.pone.0346573 (PMC13052905; doi:10.1371/journal.pone.0346573)
Supplement: S1 Table — (DOCX) [file pone.0346573.s001.docx]

**S1 Table. Complete Pearson Correlation Matrix**

| **Variables** | **1** | **2** | **3** | **4** | **5** | **6** | **7** | **8** | **9** | **10** | **11** | **12** | **13** | **14** | **15** |
| --- | --- | --- | --- | --- | --- | --- | --- | --- | --- | --- | --- | --- | --- | --- | --- |
| 1. Gender |  |  |  |  |  |  |  |  |  |  |  |  |  |  |  |
| 2. Age | ,10 |  |  |  |  |  |  |  |  |  |  |  |  |  |  |
| 3. Cheating in exams | ,10 | -,07 |  |  |  |  |  |  |  |  |  |  |  |  |  |
| 4. Plagiarism | ,06 | -,10 | ,42^***^ |  |  |  |  |  |  |  |  |  |  |  |  |
| 5. Falsification | ,07 | -,06 | ,48^***^ | ,48^***^ |  |  |  |  |  |  |  |  |  |  |  |
| 6. Open-Mindedness | -,04 | -,02 | -,03 | -,16^**^ | -,05 |  |  |  |  |  |  |  |  |  |  |
| 7. Conscientiousness | -,16^**^ | ,03 | -,06 | -,21^**^ | -,18* | -,04 |  |  |  |  |  |  |  |  |  |
| 8. Extraversion | -,01 | ,15* | -,14 | -,23^**^ | -,17* | ,31^***^ | ,25^***^ |  |  |  |  |  |  |  |  |
| 9. Agreeableness | -,09 | ,02 | -,04 | -,21^**^ | -,09 | ,08 | ,14 | ,10 |  |  |  |  |  |  |  |
| 10. Negative Emotionality | -,05 | -,15 | ,01 | ,24^**^ | ,12 | -,03 | -,29^***^ | -,18^*^ | -,29^***^ |  |  |  |  |  |  |
| 11. Machiavellianism | ,23^**^ | -,04 | ,17^**^ | ,25^***^ | ,21^**^ | ,02 | -,07 | -,03 | -,13 | ,02 |  |  |  |  |  |
| 12. Narcissism | ,23^**^ | ,02 | ,12 | ,07 | ,15 | ,35^***^ | ,01 | ,40^***^ | ,02 | -,22^**^ | ,32^***^ |  |  |  |  |
| 13. Psychopathy | ,26^***^ | ,03 | ,23^**^ | ,34^***^ | ,28^***^ | ,10 | -,31^***^ | ,08 | -,32^***^ | ,07 | ,27^***^ | ,35^***^ |  |  |  |
| 14. Sadism | ,37^***^ | -,06 | ,26^***^ | ,33^***^ | ,34^***^ | ,01 | -,26^***^ | -,08 | -,31^***^ | ,09 | ,46^***^ | ,31^***^ | ,44^***^ |  |  |
| 15. Moral disengagement | ,15 | -,16^**^ | ,24^**^ | ,30^***^ | ,17^*^ | -,31^***^ | -,12 | -,25^***^ | -,26^***^ | ,19^*^ | ,37^***^ | ,07 | ,29^***^ | ,44^***^ |  |
| *Note*: **p* < .05, ***p* < .01, ****p* < .001. | | | | | | | | | | | | | | | |
