# Supplementary material for: The role of personality traits and moral disengagement in academic dishonesty: An analysis of the big five and the dark tetrad
Source: PLoS One. 2026 Apr 6;21(4):e0346573. doi: 10.1371/journal.pone.0346573 (PMC13052905; doi:10.1371/journal.pone.0346573)
Supplement: S2 Table — (DOCX) [file pone.0346573.s002.docx]

**S2 Table. Unstandardised regression coefficients of cheating on tests predicted by socio-demographics, personality and moral disengagement.**

|  |  | **Model 1** |  |  | **Model 2** |  |  | **Model 3** |  |  | **Model 4** |  |
| --- | --- | --- | --- | --- | --- | --- | --- | --- | --- | --- | --- | --- |
| *Predictors* | *b(SE)* | *95% CI* | *p* | *b(SE)* | *95% CI* | *p* | *b(SE)* | *95% CI* | *p* | *b(SE)* | *95% CI* | *p* |
| Gender | .10(.16) | -.10-.52 | .19 | .09(.16) | -.15-.49 | .29 | -.02(.17) | -.38-.29 | .80 | -.02(.17) | -.37-.30 | .84 |
| Age | -.07(.01) | -.03-.01 | .37 | -.06(.01) | -.03-.01 | .48 | -.04(.01) | -.03-.02 | .65 | -.02(.01) | -.03-.02 | .79 |
| Education level | -.01(.04) | -.08-.07 | .90 | -.00(.04) | -.08-.08 | .95 | -.00(.04) | -.08-.08 | .99 | -.01(.04) | -.08-.07 | .90 |
| Extraversion |  |  |  | -.13(.10) | -.36-.05 | .14 | -.18(.11) | -.42-.00 | **.05** | -.16(.11) | -.40-.03 | .08 |
| Agreeableness |  |  |  | -.04(.11) | -.28-.16 | .61 | .08(.12) | -.13-.34 | .38 | .08(.12) | -.12-.35 | .35 |
| Conscientiousness |  |  |  | -.02(.09) | -.21-.16 | .79 | .07(.10) | -.11-.27 | .40 | .07(.10) | -.12-.26 | .45 |
| Negative Emotionality |  |  |  | -.09(.10) | -.30-.09 | .29 | -.01(.10) | -.21-.18 | .89 | -.02(.10) | -.23-.17 | .79 |
| Open-Mindedness |  |  |  | .01(-.10) | -.18-.21 | .87 | -.03(.11) | -.23-.17 | .75 | .01(.11) | -.20-.22 | .94 |
| Machiavellianism |  |  |  |  |  |  | .02(.11) | -.18-.25 | .78 | .07(.10) | -.22-.22 | .97 |
| Narcissism |  |  |  |  |  |  | .08(.10) | -.12-.28 | .45 | .00(.11) | -.13-.27 | .50 |
| Psychopathy |  |  |  |  |  |  | .19(.13) | -.00-.50 | **.05** | .17(.13) | -.03-.48 | .08 |
| Sadism |  |  |  |  |  |  | .18(.11) | -.03-.42 | .09 | .15(.12) | -.07-.40 | .16 |
| Moral disengagement |  |  |  |  |  |  |  |  |  | .11(.12) | -.10-.36 | .26 |
| *R^2^ / R^2^ adjusted* | .02/-.00 | | | .04/-.01 | | | .12/.05 | | | .13/.05 | | |

*Note*. *b*=beta; *SE*=Standar Error; *95% CI*=Confidence Interval, *p*=p value.
