# Supplementary material for: The role of personality traits and moral disengagement in academic dishonesty: An analysis of the big five and the dark tetrad
Source: PLoS One. 2026 Apr 6;21(4):e0346573. doi: 10.1371/journal.pone.0346573 (PMC13052905; doi:10.1371/journal.pone.0346573)
Supplement: S3 Table — (DOCX) [file pone.0346573.s003.docx]

**S3 Table. Unstandardised regression coefficients of plagiarism predicted by socio-demographics, personality and moral disengagement.**

|  |  | **Model 1** |  |  | **Model 2** |  |  | **Model 3** |  |  | **Model 4** |  |
| --- | --- | --- | --- | --- | --- | --- | --- | --- | --- | --- | --- | --- |
| *Predictors* | *b(SE)* | *95% CI* | *p* | *b(SE)* | *95% CI* | *p* | *b(SE)* | *95% CI* | *p* | *b(SE)* | *95% CI* | *p* |
| Gender | .07(.18) | -.20-.50 | .41 | .03(.17) | -.27-.42 | .66 | -.11(.17) | -.58-.10 | .16 | -.11(.17) | -.58-.10 | .16 |
| Age | -.09(.01) | -.04-.01 | .29 | -.05(.01) | -.03-.01 | .52 | -.03(.01) | -.03-.02 | .70 | -.03(.01) | -.03-.02 | .69 |
| Education level | -.06(.05) | -.12-.06 | .48 | -.03(.04) | -.10-.07 | .68 | -.02(.04) | -.09-.07 | .83 | -.01(.04) | -.09-.07 | .84 |
| Extraversion |  |  |  | -.12(.11) | -.38-.06 | .15 | -.19(.11) | -.47-.04 | **.02** | -.19(.11) | -.48-.03 | **.02** |
| Agreeableness |  |  |  | -.12(.12) | -.43-.05 | .13 | .02(.12) | -.21-.27 | .82 | .02(.12) | -.21-.27 | .82 |
| Conscientiousness |  |  |  | -.11(.10) | -.34-.06 | .17 | -.00(.10) | -.20-.19 | .98 | -.00(.10) | -.20-.19 | .99 |
| Negative Emotionality |  |  |  | -.13(.09) | -.04-.33 | .12 | .18(.09) | -.03-.38 | **.02** | .18(.09) | .03-.38 | **.02** |
| Open-Mindedness |  |  |  | .12(.11) | -.37-.05 | .14 | -.17(.10) | -.42-.02 | **.03** | -.17(.11) | -.44-.01 | **.04** |
| Machiavellianism |  |  |  |  |  |  | .10(.11) | -.08-.36 | .22 | .10(.11) | -.09-.37 | .23 |
| Narcissism |  |  |  |  |  |  | .10(.11) | -.10-.32 | .30 | .10(.11) | -.10-.32 | .30 |
| Psychopathy |  |  |  |  |  |  | .27(.13) | -.15-.66 | **.00** | .27(.13) | -.14-.66 | .**00** |
| Sadism |  |  |  |  |  |  | .15(.12) | -.04-.42 | .11 | .15(.12) | -.05-.42 | .11 |
| Moral disengagement |  |  |  |  |  |  |  |  |  | -.01(.12) | -.25-.22 | .90 |
| *R^2^ / R^2^ adjusted* | .02/-.00 | | | .14/.09 | | | .28/.22 | | | .28/.22 | | |

*Note*. *b*=beta; *SE*=Standar Error; *95% CI*=Confidence Interval, *p*=p value.
