# Supplementary material for: The role of personality traits and moral disengagement in academic dishonesty: An analysis of the big five and the dark tetrad
Source: PLoS One. 2026 Apr 6;21(4):e0346573. doi: 10.1371/journal.pone.0346573 (PMC13052905; doi:10.1371/journal.pone.0346573)
Supplement: S4 Table — (DOCX) [file pone.0346573.s004.docx]

**S4 Table. Unstandardised regression coefficients of falsification predicted by socio-demographics, personality and moral disengagement.**

|  |  | **Model 1** |  |  | **Model 2** |  |  | **Model 3** |  |  | **Model 4** |  |
| --- | --- | --- | --- | --- | --- | --- | --- | --- | --- | --- | --- | --- |
| *Predictors* | *b(SE)* | *95% CI* | *p* | *b(SE)* | *95% CI* | *p* | *b(SE)* | *95% CI* | *p* | *b(SE)* | *95% CI* | *p* |
| Gender | .08(.11) | -.11-.33 | .33 | .05(.11) | -.15-.30 | .51 | -.10(.12) | -.37-.10 | .23 | -.10(.12) | -.37-.10 | .21 |
| Age | -.05(.01) | -.02-.01 | .56 | -.03(.01) | -.02-.01 | .76 | .01(.01) | -.01-.02 | .92 | -.00(.01) | -.02-.02 | .96 |
| Education level | -.07(.03) | -.08-.03 | .39 | -.05(.03) | -.07-.04 | .57 | -.05(.03) | -.07-.04 | .53 | -.04(.03) | -.07-.04 | .60 |
| Extraversion |  |  |  | -.11(.07) | -.23-.05 | .21 | -.18(.07) | -.29-.00 | **.04** | -.19(.07) | -.31-.01 | **.03** |
| Agreeableness |  |  |  | -.05(.08) | -.20-.12 | .59 | .09(.08) | -.07-.25 | .30 | .08(.08) | -.08-.24 | .32 |
| Conscientiousness |  |  |  | -.12(.07) | -.22-.04 | .17 | -.01(.07) | -.14-.12 | .89 | -.00(.07) | -.14-.13 | .95 |
| Negative Emotionality |  |  |  | .05(.06) | -.09-.15 | .60 | .10(.06) | -.04-.19 | .24 | .11(.06) | -.04-.20 | .19 |
| Open-Mindedness |  |  |  | .02(.07) | -.16-.12 | .81 | -.08(.07) | -.20-.07 | .33 | -.11(.07) | -.23-.05 | .21 |
| Machiavellianism |  |  |  |  |  |  | .02(.07) | -.13-.16 | .82 | .04(.08) | -.12-.18 | .67 |
| Narcissism |  |  |  |  |  |  | .14(.07) | -.03-.25 | .14 | .15(.07) | -.03-.25 | .12 |
| Psychopathy |  |  |  |  |  |  | .18(.09) | -.00-.34 | **.05** | .19(.09) | .01-.36 | .**04** |
| Sadism |  |  |  |  |  |  | .25(.08) | .04-.36 | .**01** | .27(.08) | .06-.38 | .**01** |
| Moral disengagement |  |  |  |  |  |  |  |  |  | -.10(.08) | -.24-.08 | .31 |
| *R^2^ / R^2^ adjusted* | .01/-.00 | | | .06/.01 | | | .20/.13 | | | .20/.13 | | |

*Note*. *b*=beta; *SE*=Standar Error; *95% CI*=Confidence Interval, *p*=p value.
